# A SIRT1-independent mechanism mediates protection against steroid-induced senescence by resveralogues in equine tenocytes

**DOI:** 10.1371/journal.pone.0309301

**Published:** 2024-08-22

**Authors:** Neda Heidari, Richard G. A. Faragher, Graham Pattison, Jayesh Dudhia, Roger K. W. Smith

**Affiliations:** 1 Department of Clinical Sciences and Services, The Royal Veterinary College of University of London, North Mymms, Hertfordshire, United Kingdom; 2 School of Applied Sciences, University of Brighton, Brighton, East Sussex, United Kingdom; 3 School of Chemistry, College of Health and Science, University of Lincoln, Lincoln, United Kingdom; Weizmann Institute of Science, ISRAEL

## Abstract

Tendinopathy is a common age-related disease which causes significant morbidity for both human athletes and performance horses. In the latter, the superficial digital flexor tendon is an excellent model for human tendinopathies because it is a functional homologue of the human Achilles tendon and a primary site of injuries with strong similarities to the human disease. Corticosteroids have been previously used clinically to treat tendinopathic inflammation, but they upregulate the p53-p21 axis with concomitant reductions in cell proliferation and collagen synthesis in human tenocytes. This phenotype is consistent with the induction of cellular senescence *in vitro* and *in vivo* and probably represents an important clinical barrier to their effective use. Because of the many differences in senescence mechanisms between species, this study aimed to evaluate these mechanisms after corticosteroid treatment in equine tenocytes. Exposure to clinically reflective levels of dexamethasone for 48 hours drove equine tenocytes into steroid induced senescence (SIS). This was characterised by permanent growth arrest and upregulation of p53, the cyclin dependent kinase inhibitors p21^waf^ and p16^ink4a^ as well as the matrix degrading enzymes MMP1, MMP2 and MMP13. SIS also induced a distinctive equine senescence associated secretory phenotype (eSASP) characterised by enhanced secretion of IL-8 and MCP-1. Preincubation with resveratrol or the potent SIRT1 activator SRT1720 prevented SIS in equine tenocytes, while treatment with the non-SIRT1 activating resveratrol analogue V29 was equally protective against SIS, consistent with a novel, as yet uncharacterised SIRT1-indendent mechanism which has relevance for the development of future preventative and therapeutic strategies.

## Introduction

Tendinopathy is a common injury in both human and equine athletes with significant impact on healthcare systems. The equine superficial digital flexor tendon (SDFT) is an important site of tendinopathy in its own right and also a highly relevant model for studying human tendon injuries because of its functional homology to the Achilles tendon. The SDFT displays many similarities to the human Achilles in physiology, pathogenesis and subsequent adverse consequences of injury.

Recently, the inflammatory cascade, and, in particular, its resolution, has been shown to be important in the healing of tendinopathy [1–3]. In the past, a common approach to the management of tendinopathies in humans involved the administration of corticosteroids. Unfortunately, although these steroids have powerful anti-inflammatory effects, treatment of cultured human tenocytes with nanomolar concentrations of dexamethasone for as little as 24 hours induces a state of cellular senescence [4]. Exposure for 72 hours *in vitro* drives more than 60% of cells in the population into senescence and these results have been replicated by subacromial injection of the glucocorticoid Depo-Medrone in patients with rotator cuff tendinopathy [4]. At one-week post injection the number of senescent cells present in the tendon *in vivo* was increased 4-fold compared with biopsies taken immediately prior to injection.

Senescent cells are the living, but permanently non-dividing, forms of cells which are normally capable of division *in vivo* [5]. Senescence is distinct from both quiescence and, in systems in which it is possible to experimentally separate the two processes, from terminal differentiation. Entry into a senescent state is associated with a sequence of temporal changes in gene expression and a profound change in phenotype. This occurs for three primary purposes; firstly, to prevent the cell from dividing, next to shift it into a catabolic phenotype, and finally to facilitate the removal of the cell by the immune system. In the best-studied model system (human fibroblasts), senescent cells are prevented from dividing by overexpression of the cyclin dependent kinase inhibitors p21^waf^ and p16^ink4a^, typically as a result of the activation of p53. Then, a wide range of molecules similar to those upregulated in the early remodelling phase of wound repair are over-expressed. Known collectively as the Senescence Associated Secretory Phenotype (or SASP) these include both inflammatory cytokines (e.g. IL-1β, IL-6, IL-8 and MCP-1), matrix remodelling enzymes (e.g. MMP1, 2, 9 and 13), and growth factors (e.g. PDGF-AA, Gro and scatter factor). Cell surface molecules (e.g. ICAM and uPAR/CD87) are also up-regulated, especially the natural killer group 2D (NKG2D) ligands. These ligands are not widely expressed on division competent cells allowing for the specific recognition, interaction and elimination of senescent cells by the immune system [5].

Due to increased production of senescent cells over time, and a progressive failure of immune clearance, senescent cells accumulate in many mammalian tissues where they are known to be a primary cause of ageing and pathology [6]. In equine tendon, changes associated with ageing and exercise mirror the SASP [7] and so we hypothesise that the problems associated with tendon integrity following repeated dexamethasone injection result from the induction of senescence as a side-effect (a phenomenon referred to as steroid induced senescence (SIS)) which could also provide a preventative strategy for naturally-occurring tendinopathy.

For human tenocytes *in vitro*, it has been shown that treatment with resveratrol (*trans*-3,5,4’-trihydroxystilbene), which is known to activate the NAD^+^-dependent deacylase Sirtuin 1 (SIRT1), is protective against SIS [4]. SIRT1 is down-regulated by dexamethasone treatment in tenocytes and overexpression of the molecule is also protective against SIS in human tenocytes. Taken together this suggests a model in which steroid treatment reduces active SIRT1 levels which, in turn, leads to an accumulation of acetylated p53 and entry into senescence. However, stilbenes, such as resveratrol, activate a wide variety of pathways, not simply SIRT1, which has, until recently, hampered a precise description of their mechanism of action. To overcome this, we developed a simple and convenient one-pot method for the synthesis of these molecules using sequential Michaelis-Arbuzov rearrangement and Horner- Wadsworth- Emmons reactions [8], which gave ready access to a series of ~50 novel molecules, known as resveralogues. Using these, we have demonstrated that it is possible to rescue multiple types of human cells from senescence using either resveratrol or resveralogues that have no capacity to activate SIRT1 at all, indicating that a separate, SIRT1-independent pathway can also mediate rescue from the senescent state [9].

Although large and long-lived animals typically show significant overlap in the molecular pathways used to regulate cellular senescence, important species and tissue differences are common in the response to specific senescence inducing stimuli and cell senescence mechanisms in equids remain poorly understood [10]. Therefore the aims of this study was to induce SIS in equine tenocytes and investigate whether resveratrol and specific analogues against key mechanisms can block the SIS.

## Methods

### Cell isolation and culture condition

This study used the equine super digital flexor tendon (SDFT) as a source for tenocyte isolation as previously described [11]. Briefly, SDFT were harvested from 3 three adult horses (aged between 2 and 11 years old, male). For digestion, samples of SDFTs from the mid metacarpal region and free of loose connective tissue were diced into approximately 5 mm^3^ pieces and washed with phosphate-buffered saline (PBS) (Sigma, UK) containing 1% (v/v) penicillin/streptomycin P/S, (Thermo Fisher, UK). The tendon pieces (approximately 2 g) were placed in 20 mL high glucose (4.5 g/L) Dulbecco’s modified Eagles’ medium (DMEM) (Thermo Fisher, UK) containing 1mg/ml type 2 collagenase (Worthington Biochemical corporation, UK) and were incubated at 37°C for 6 hours with gentle shaking on an ORBI- plate SHAKER^**™**^JR (Benchmark Scientific, UK)., The digestion solution was passed through a cell filter (70 μM; BD Bioscience) and centrifuged (350 RCF, 5 min, 21°C). The cell pellet was washed in PBS and resuspended in complete medium consisting of DMEM containing 4.5 g/L glucose and supplemented with 10% (v/v) foetal bovine serum (FBS) (Thermo Fisher, UK), and 1% P/S (v/v). Tenocytes were seeded into culture flasks (Fisher, UK) at a density of 100,000 cells/cm^-2^ and were cultured in a humidified incubator with 5% CO_2_ at 37°C. The culture medium was changed every two days. Cells were passaged at 70% confluency by Trypsin-EDTA (Thermo Fisher 25200056, UK) dispersion and seeded at 10,000 cells/cm^-2^ in T75 flasks for further expansion and passage as necessary. Cells were resuspended in cell freezing medium (CellBanker 2, AMSBio) and stored in liquid nitrogen until used for experiments.

### Treatment with dexamethasone, resveratrol and resveralogues

Tenocytes were seeded in 12 well tissue culture plates at a concentration of 1x10^5^ cells/well in the complete medium and allowed to attach at 37°C and 5% CO_2_ for 24 h before treatment. Cells were treated with complete medium supplemented with dexamethasone (Sigma, UK) at 1 and 10 μM for 48 h. In additional experiments, tenocytes were co-treated in media containing resveralogues (either 2μM resveratrol (3,4’,5-trihydroxystilbene, Abcam, UK), 10μM V29 (((E)-N-(4-(3,5-dimethoxystyryl) phenyl)methanesulfonamide) Merck, UK) or V34 (((E)-5-(4-(3,5-dimethoxystyryl)phenyl)-1H–tetrazole) [12] with dexamethasone at 1 and 10 μM for 48 h. Control cultures were incubated with complete media alone. After this, the cultures were washed with PBS once and allowed to recover in growth medium overnight. Ethanol was used to produce stock solution of dexamethasone and Dimethyl sulfoxide (DMSO) (Thermofisher, UK) was used to prepare stock solutions of resveratrol and resveralogues.

Tenocytes treated with 10 μM etoposide (Abcam, UK) for 48 h to induce replicative senescence were used as positive controls. After treatment, the cultures were washed with PBS and allowed to recover with a complete medium overnight before use.

Tenocytes seeded as above were co-treated in complete media containing SRT-1720 (0.5 and 1μM) (Abcam, UK) and dexamethasone at 1 and 10 μM for 48 h. Control groups were incubated in the complete medium only.

### Effect of dexamethasone on quiescent tenocytes

Tenocytes at passage number 4 were seeded at 10,000 cells/cm^-2^ in T25 flasks in complete medium. After 24 h, the medium was replaced with serum-free DMEM containing 1 and 10 μM concentrations of dexamethasone for 48h. The control cells were kept in serum-free DMEM only. After treatment, cells were detached and then plated on 12 well plates for 48h in the complete medium.

### Cell viability assay

Cell viability was measured by thiazolyl blue tetrazolium bromide (MTT) (Abcam, UK) assay 24h after removing dexamethasone and etoposide treatment. Cultures were washed twice with PBS. A standard curve was used to establish a linear relationship between cell concentration and MTT absorbance values. To measure cell viability tenocytes were seeded into the wells of 96 well plates at a concentration of 5×10^5^ cells/Well in duplicates. To optimise the cell seeding density for the MTT assay, a series of concentrations in 100 μL medium including 5 ×10^5^,2.5×10^5^,1.25× 10^5^, 6.3× 10^4^, 3.1× 10^4^, 1.6x10^4^, 8× 10^3^ cell/well were set up by serial dilution in the wells of 96-well plate (Thermo Fisher, UK), and incubated for 24 hours. After 24 hours of incubation, 10μL of MTT solution (5mg/mL) was added to each well and incubated at 37°C (5% CO_2_) for 2 hours until purple-coloured formazan product developed. At the end of the incubation, the medium was removed from each well and 100μL of DMSO (Thermofisher, UK) was added to each well to lyse the cells. The plate (Thermo Fisher, UK) was then left for 5 minutes in a plate shaker and the absorbance was measured by spectrophotometry at a wavelength of 570 nm.

### Determination of culture growth fraction by EdU incorporation

The Click-iT EdU Alexa Fluor 555 imaging kits (Thermofisher C10337, UK) were used to determine S phase traverse in accordance with manufacturer’s protocol. Briefly, tenocytes were incubated with 10 μM 5-ethynyl-2’-deoxyuridine (EdU) for 3 hours. Following the EdU incubation, cells were rinsed with medium and fixed using 3.7% (v/v) formaldehyde for 10 minutes. Fixed cells were permeabilised using a solution of 0.5% (v/v) Triton-X-100 in PBS for 20 minutes. After permeabilisation, cells were washed twice with PBS and treated with 50μL of the Click-iT reaction cocktail for 30 minutes. The cells were then washed once with PBS and counterstained with 1 μg mL^-1^ DAPI in PBS for 10 min. Fluorescence was measured using a fluorescent microscopy (EVOS FL, Thermofisher, UK). To determine the proliferating fraction, either a minimum of 500 total nuclei were counted in random fields on each of three wells. Experiments were repeated in triplicate.

### Determination of growth fraction by Ki67 immunostaining

After removing dexamethasone, cells were washed twice in 1mL of PBS and fixed with 3.7% formaldehyde solution v/v (Sigma, UK) for 10 minutes at room temperature, washed twice with wash buffer (3% BSA in PBS, w/v) and then permeabilised with 0.5% Triton^®^ X-100 (Triton, Sigma) v/v in PBS. After three PBS washes, the cells were incubated with 20 μg/mL Alexa Fluor^®^ 488 Anti-Ki67 antibody (Abcam, UK) at room temperature for 4 hours. The nuclei stained with diamidino-2-phenylindole (DAPI) (Sigma, UK) and visualised by fluorescent microscopy (EVOS FL, Thermofisher, UK). The percentage of Ki67-positive cells was quantified by counting the number of Ki67-positive cells as a proportion of total DAPI-positive cells. A minimum of 500 nuclei were counted in randomly selected fields. Experiments were repeated in triplicate.

### Quantification of senescence markers by real time PCR

Tenocytes were seeded onto 6-well tissue culture plates at a concentration of 2×10^5^ cells/well in growth media and allowed to attach at 37°C and 5% CO^2^ for 24 h. Complete medium supplemented with dexamethasone at 1 and 10 μM concentrations were added to cells and incubated for 48 h. Control cells were grown in media only. After 48 h incubation, cells were washed with PBS twice and incubated in medium for 24, 48 and 96 h. The total RNA was extracted from cells using Tri-reagent (Sigma, UK) followed by RNeasy mini kit (Qiagen, Manchester, UK). The total RNA concentration and purity were measured by 260/280 nm absorbance ratio (Nanodrop, DeNovix,Cambridge Bioscience). cDNA was synthesised from 1 μg of RNA using the sensiFAST cDNA synthesis kit (Bioline, London, UK). Primers (Table 1) were designed using primer3 (http://primer3.ut.ee/) for synthesis (Sigma-Aldrich). 2 μl aliquots of template cDNA were used for qPCR (Bioline SeniMix No-ROX, Bioscience, UK) using the Biorad C1000 Touch Thermal Cycler (Biorad, Hertfordshire, UK). Reactions were performed in duplicate. PCR initial activation was done at 95°C for 10 min, followed by 44 cycles of 95°C for 15 seconds, 60°C for 15 seconds and 72°C for 15 seconds. At the end of the program, a melt curve was produced by taking readings every 0.5°C from 60°C to 90°C. GAPDH was used as an internal reference. Relative gene expression levels were calculated using the 2^-ΔΔCT^ method (ref). A one-way ANOVA with post hoc Tukey test was used to determine significant differences in the mean gene expression using three biological replicates.

**Table 1:** The equine primer sequences used for real-time PCR.

Gene	Protein name	Forward primer	Reverse primer
GAPDH	Glyceraldehyde 3-phosphate dehydrogenase	CGACCACTTTGTCAAGCTCA	GTCCACCACCCTATTGCTGT
TP53	Tumor protein P53	GGCAGTCTACCTCCTCCCAT	TACCCGAAAATGCCAGGGA
CDKN1A	Cyclin-dependent kinase inhibitor 1 (P21)	ACATACTCTGCTTGCCACCC	GGCCCCCTTCAAAGTGCTAT
CDKN2A	Cyclin-dependent kinase inhibitor 2A (P16)	CCGGAGACACTTCGACACAT	GGTGGGTTCTCCCTCCTGAA
MMP1	Matrix metallopeptidase 1 (interstitial collagenase)	CTTTGATGGACCTGGAGGAA	GAATGGCCAAATTCATGAGC
MMP2	Matrix metallopeptidase 2 (72 kDa type IV collagenase)	CAGGAGGAGAAGGCTGTGTT	AGGGTGCTGGCTGAGTAGAC
MMP13	Matrix metallopeptidase 13 (collagenase 3)	GCCACTTTGTGCTTCCTGAT	CGCATTTGTCTGGTGTTTTG

### Cytokine array

Tenocytes were seeded in a 6-well plate at a final concentration of 5×10^5^ cells/Well. They were treated with dexamethasone for 48 h. The medium then was removed from each well and replaced with complete medium for 5 days. The media were collected and immediately frozen and stored at -20°C until analysis. RayBio^®^ C-Series Equine Cytokine Array 1 (RayBiotech, UK) was used for the semi-quantitative detection of IL-8, MCP-1, VEGF-A, IL-2, IL-4, IL-15, IL-10, IL-1Ra, IL-alpha and IFN-gamma in accordance with the manufacturer’s protocol. Control cultures were maintained with complete media alone.

### SA-β-Gal staining

The senescence-associated beta-galactosidase (SA-β-Gal) activity in tenocytes treated with dexamethasone and resveratrol, was measured 7 days after removing treatment using the Senescence β-Galactosidase Staining Kit (Cell Signaling Technology, USA), following the manufacturer’s instructions. Cells were examined via a light microscope, and the percentage of SA-β-gal-positive cells was calculated.

### Data and statistical analysis

All data were analysed using Graphpad Prism 9 software. Data values are displayed as the mean ± standard deviation, obtained from three distinct biological samples (from 3 horses). Statistical analyses were performed using one or two way ANOVA with Bonferroni‘s multiple comparison test. Significance was detected at p-value of <0.05 (*), <0.01 (**), <0.001(***).

## Results

### Treatment with dexamethasone rapidly induces a senescent state characterised by growth arrest, upregulation of cyclin-dependent kinase inhibitors (CDKIs), SASP and MMPs

To treat tendinopathy, usually, 4 to 40 mg (equivalent to 10–100 mM) of dexamethasone is administrated directly into the tendon sheath. It is complicated to assess and measure the dexamethasone concentration in exposed tenocytes after local administration because it is associated with tissue permeability and the proximity of the tenocyte to the injection site. Approximately 1 and 10 μM systemic concentrations have been observed following systemic administration [4]. Therefore, in this study, tenocytes were cultured for 48 hours in media containing dexamethasone at 1 and 10 μM concentrations. As a positive control, tenocytes were treated for 48 hours with the DNA-damaging agent etoposide at 10 μM concentration.

**Fig 1:**
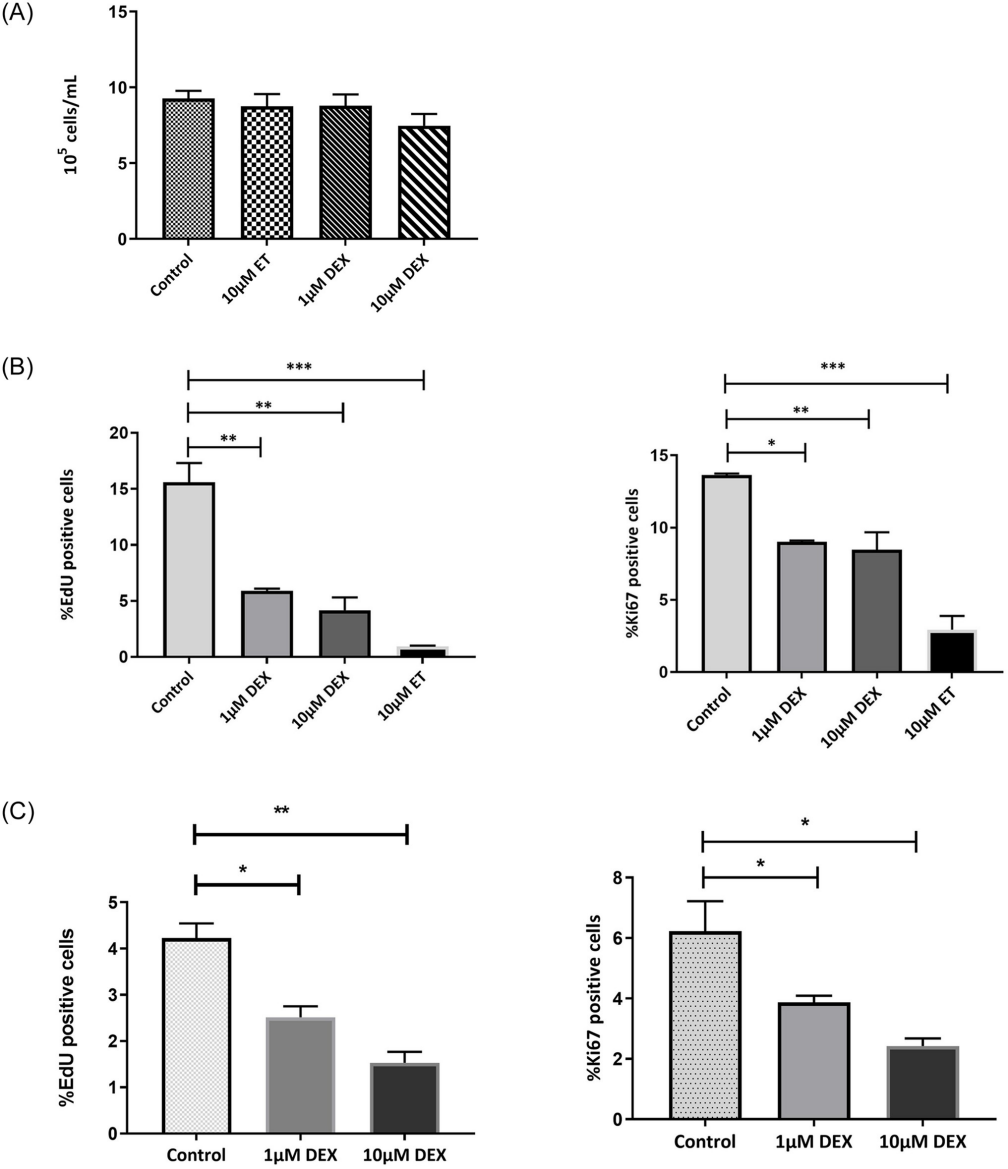
**A.** Viability of tenocytes following dexamethasone treatment by the MTT assay. The results are expressed as the mean ± SD of three independent experiments. One-Way ANOVA with Bonferroni’s Multiple Comparison Test was applied. **B.** Cell proliferation assay following dexamethasone treatment. The percentage of cell cycling after 48 h dexamethasone and etoposide treatment was measured by EdU and Ki67 labelling. The data represent the average and standard deviation of three independent experiments (n = 3 horses) using one-way ANOVA Bonferroni’s multiple comparison test. (ET: etoposide, DEX: dexamethasone) (*p<0.05, **p<0.01, ***p<0.001). **C.** Cell proliferation assay in serum free medium containing dexamethasone for 48h. The results are expressed as the Mean ± SD of three experiments using One-Way ANOVA with Turkey’s Multiple Comparison Test. EdU and Ki67 labelling were performed in duplicate and at least 500 cells were inspected to determine the percentage of ki67-positive cells(DEX: dexamethasone) (*p<0.05, **p< 0.01).

Cell viability, as measured by the MTT assay, 24 hours after removing treatment was 9.3 x 10^5^ in control cells, 8.8 x 10^5^ and 7.5 x 10^5^ in 1 and 10 μM dexamethasone-treated cells respectively and 8.7 x 10^5^ in 10 μM etoposide-treated cells (Fig 1A). Exposure to dexamethasone at both concentrations and 10 μM etoposide had no statistically significant effect on tenocyte viability compared to the control. Following dexamethasone and etoposide treatments, the total cycling fraction of cells was measured by EdU label incorporation and the percentage of EdU positive cells reduced significantly from 15.59%±1.7 to 5.9%±0.18 and 4.1%±1.12 in 1 and 10μM dexamethasone-treated cells (p = 0.01 and p = 0.001), respectively and 0.95%±0.06 in 10μM etoposide-treated cells (Fig 1B). A similar reduction was observed by Ki67 immunostaining and 9%±0.08 and 8.5%±1.2 of 1 and 10μM dexamethasone-treated cells respectively were stained positively with Ki67 compared to control (14%±0.11) (Fig 1B).

Reversibility is a defining feature of quiescent cells. Toassess whether dexamethasone-induced cell cycle arrest is reversible, tenocytes treated with dexamethasone and untreated cells were cultured in serum-free medium for 48 hours. Subsequently, cells were trypsinised and grown in complete medium for another 48 hours. As depicted in Fig 1C, the percentage of EdU-positive cells exhibited a significant reduction in both 1 and 10 μM dexamethasone-treated groups (p < 0.05 and p < 0.01, respectively) compared to the control group. This finding suggests that the non-cycling fraction of cells failed to re-enter the cell cycle even after transitioning from serum-free to complete medium. Conversely, control cells demonstrated recovery from the quiescent state upon medium replacement. A similar significant reduction in the percentage of Ki67-positive cells was observed after both concentrations of dexamethasone (p<0.05, Fig 1C). These results suggest that dexamethasone-induced cell cycle arrest in tenocytes is not readily reversible, as evidenced by the sustained reduction in proliferation markers even upon growing in complete culture condition.

#### Effect of dexamethasone on senescence markers gene expression

**Fig 2:**
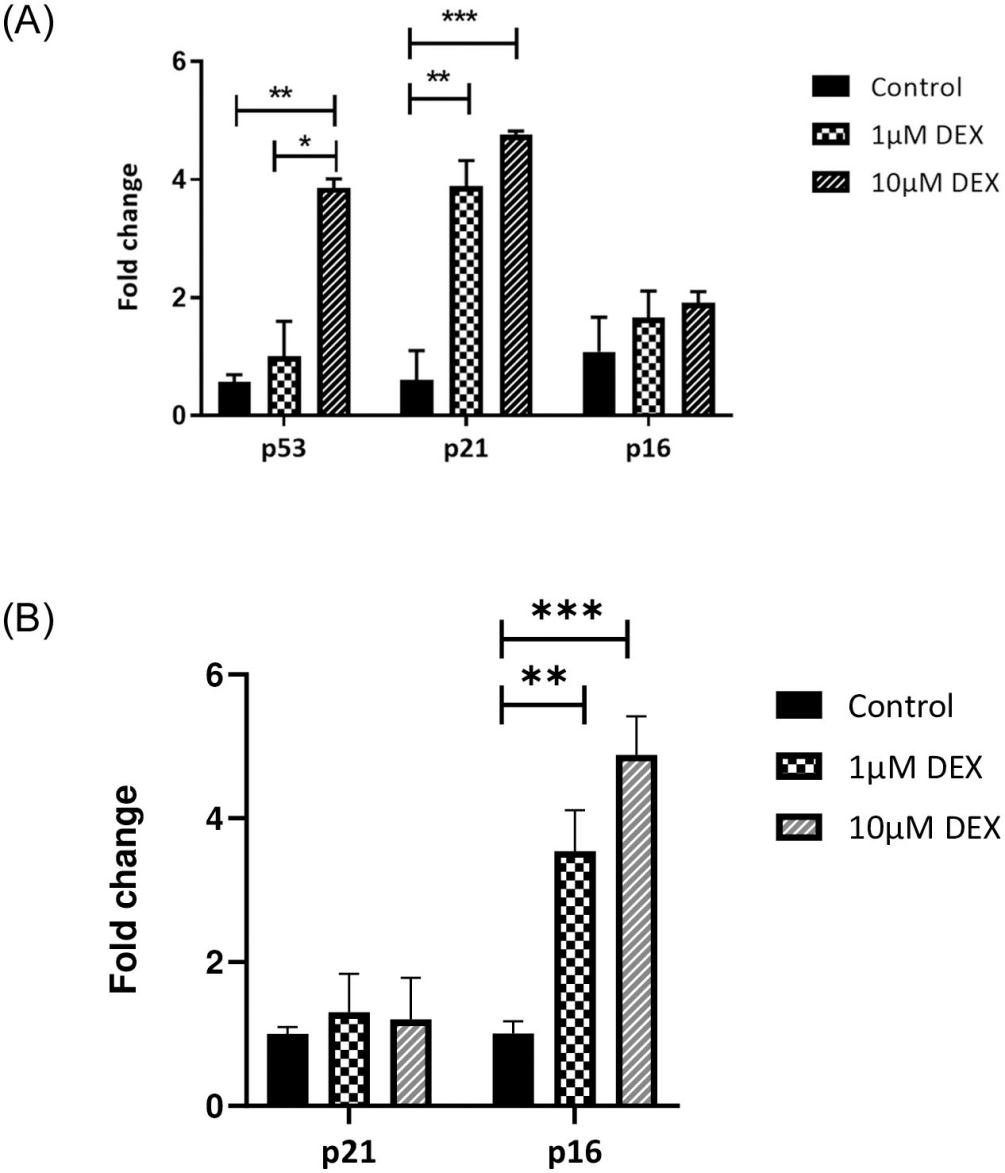
Expression of p53, p21 and p16 in dexamethasone-treated tendon derived cells. Total RNA from TDCs was analysed by qPCR at (2A) 24h and (2B) 72h following dexamethasone (DEX) treatment. P53 was not detected at 72 h (*p<0.05, **p<0.01, ***p<0.001).

P53, p21 and p16, are established markers of a senescent state. Therefore, their expression at the message level in tenocytes were measured using qPCR following 24 and 72h post-treatment with dexamethasone. Fig 2A shows the expression of these markers in tenocytes. There was a significant difference in p21 expression level between the control and 1μM (p< 0.01) and 10 μM (p<0.001) dexamethasone-treated groups (Fig 2A). The p53 gene expression in the 10 μM dexamethasone-treated group was significantly higher compared to control (p< 0.01) and 1 μM- treated cells with dexamethasone (p<0.05, Fig 2A). There was no significant difference in p53 expression at 1 μM dexamethasone. p16 gene expression was not significantly different between the treated and non-treated groups at 24 hours but was highly expressed in 1 and 10 μM dexamethasone-treated cells (p<0.01, p<0.001 respectively) by 72 hours post-treatment. (Fig 2B). These changes are very similar to those observed in human tenocytes which have entered SIS.

#### Effect of dexamethasone on SASP induction

**Fig 3:**
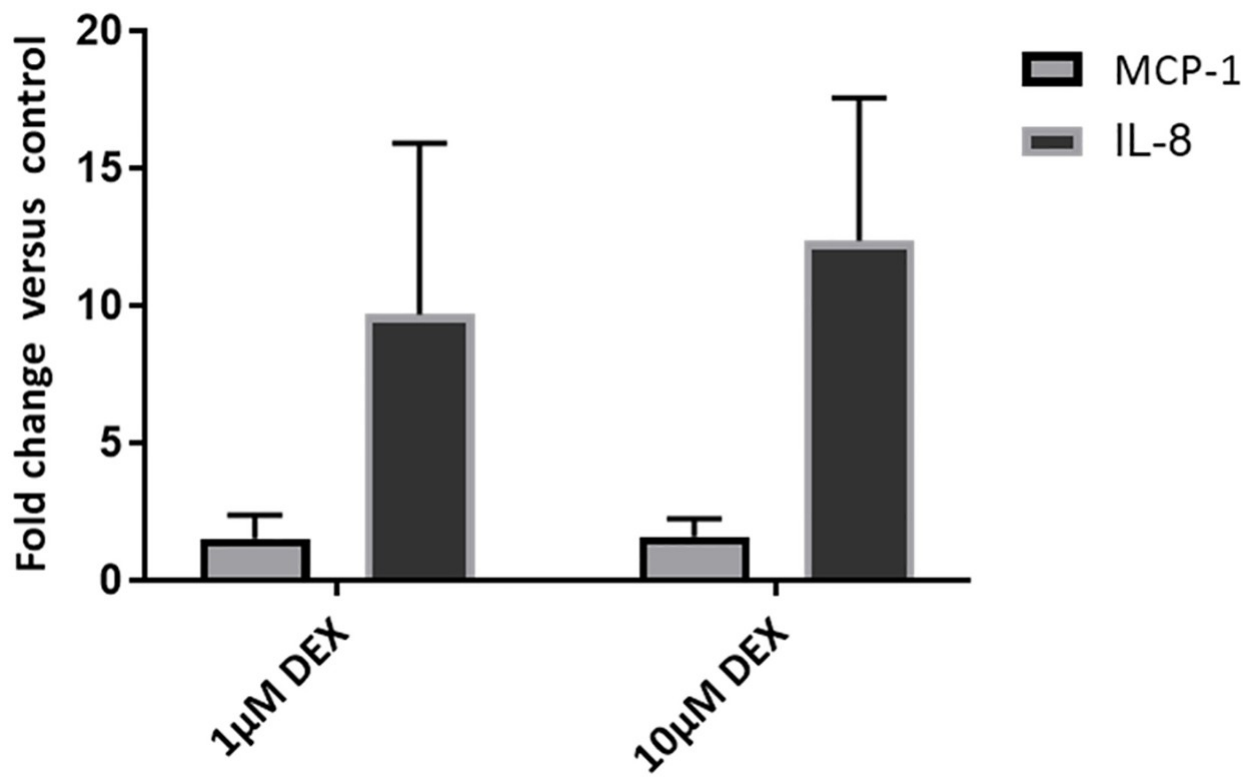
Induction of a SASP in tenocytes by dexamethasone. Cytokines were assayed in culture medium by ELISA at 5 days after dexamethasone treatment. There were no statically significant differences in IL-8 and MCP-1 levels between the 1 and 10μM concentrations of dexamethasone (DEX).

In order to determine if dexamethasone induces equine tenocytes to secrete inflammatory mediators the levels of a number of key pro-inflammatory cytokines were determined in the culture media of dexamethasone-treated tenocytes. Dexamethasone significantly elevated the SASP-associated chemokine IL-8 and monocyte chemoattractant protein-1 (MCP-1) at both 1 and 10 μM (Fig 3). Other cytokines were not expressed in equine tenocytes.

#### Effect of dexamethasone on MMP gene expression

**Fig 4:**
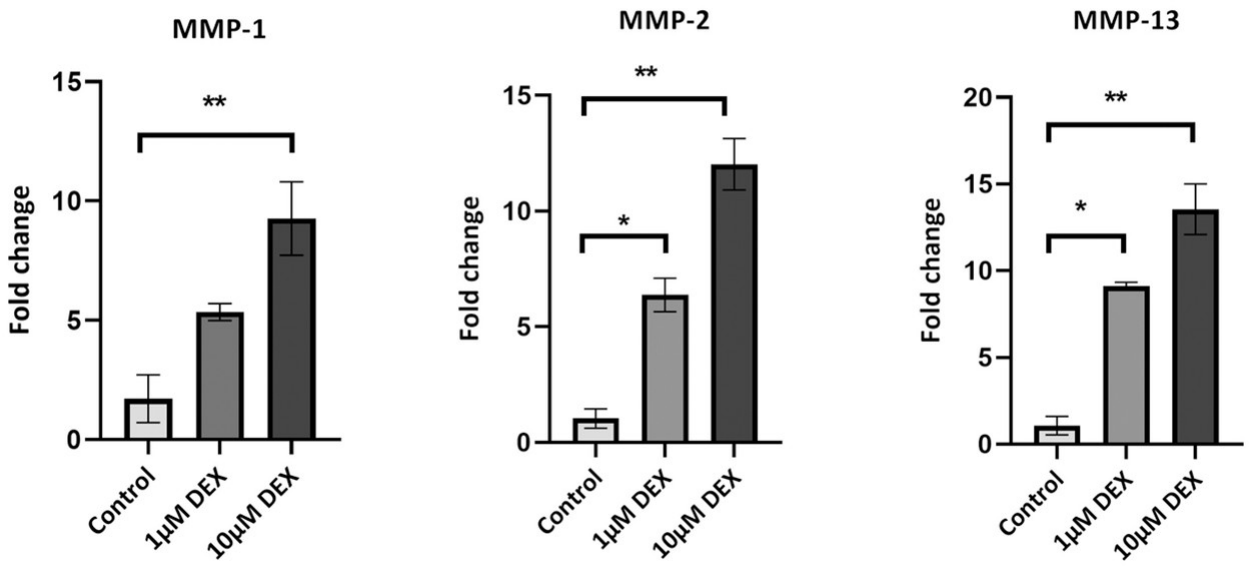
MMPs gene expression. The gene expression level of MMP-1, MMP-2 and MMP-13 in dexamethasone-treated TDCs 5 days after removing dexamethasone (DEX) was analysed by qPCR. Data were normalized to GAPDH and expressed as fold change over control levels. One-way ANOVA,* p<0.05,** p<0.01.

The expression of MMP-1, MMP-2 and MMP-13 were measured in tenocytes 5 days post treatment with 1 μM or 10 μM dexamethasone. MMP-1, MMP-2 and MMP-13 levels significantly increased after treatment with 10μM dexamethasone (p<0.01) compared to the untreated controls (Fig 4). Although no statistically significant differences were seen in MMP-1 levels between control and 1μM dexamethasone-treated tenocytes MMP-2 and MMP-13 genes were significantly upregulated post treatment (p<0.05).

### Resveratrol protects against steroid-induced senescence and blocks the SASP

**Fig 5:**
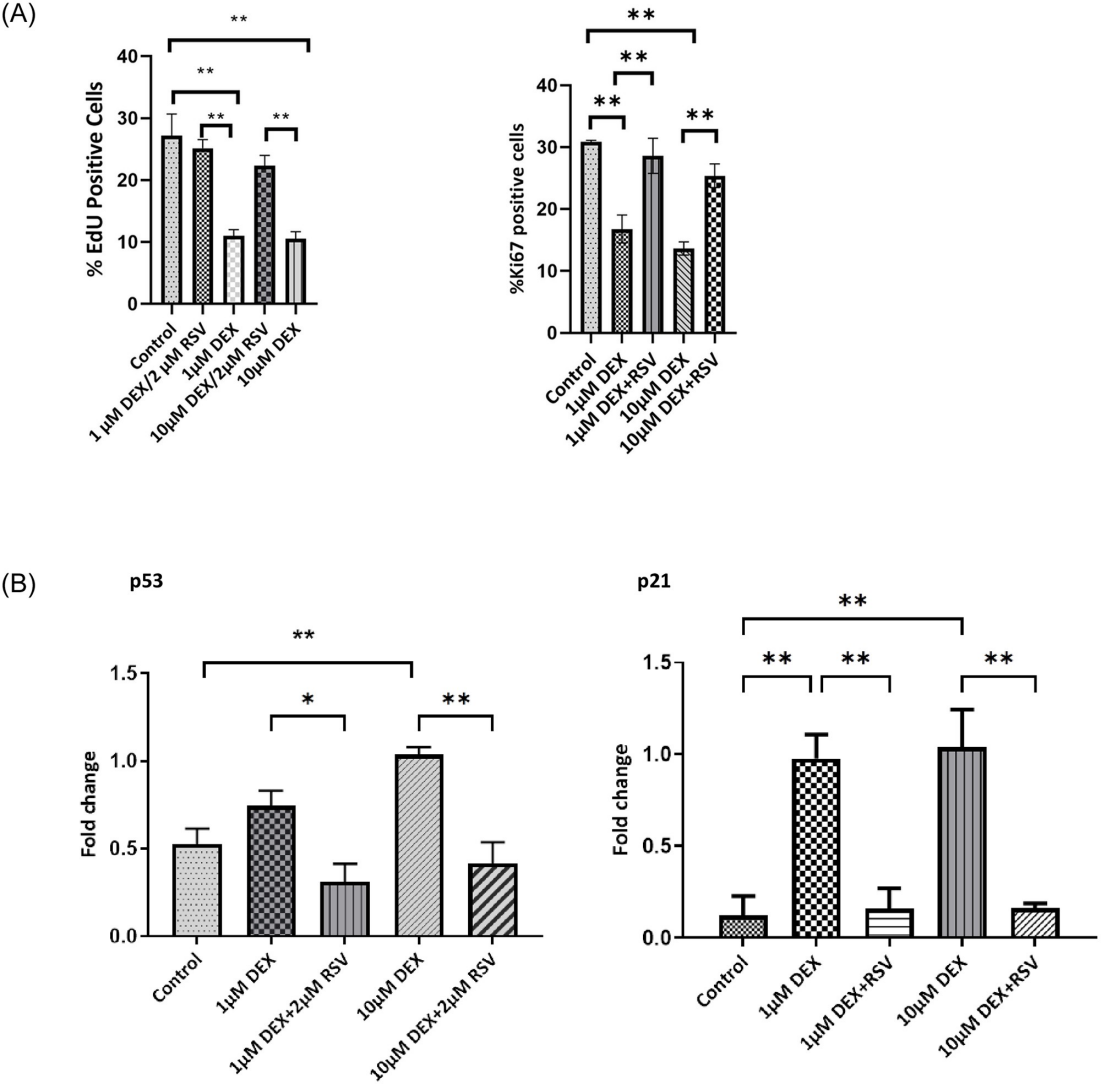
**A.** EdU and Ki67 labelling for proliferating cells. Statistically significant differences between 1 or 10μM dexamethasone (DEX) and Resveratrol (RSV)+ DEX are indicated by ** p<0.01. **B**. p53 and p21 genes expression. The expression level of p53 and p21 in dexamethasone and resveratrol-treated(DEX+RSV) TDCs 24h after removing DEX was analysed by qPCR. *p<0.05, **p<0.01.

To determine whether equine SIS can be prevented, tenocytes were treated with 1 and 10 μM dexamethasone supplemented with 2μM resveratrol for 48 h. As in previous experiments, dexamethasone-treated cells not treated with resveratrol entered into senescence as measured by the suppression of EdU incorporation (Fig 5A). However, cultures treated with resveratrol were completely protected from senescence induced by 48 hours dexamethasone treatment (Fig 5A; p<0.01 vs dexamethasone-treated cultures; no statistical difference to untreated controls). Also, the total fraction of cycling cells, as measured by pKi67 staining, showed a highly significant reduction on exposure to dexamethasone treatment at both concentrations. This was again completely prevented by supplementation of the medium with resveratrol (15.8±1.2% and 15.7±1.9% positive nuclei in cultures exposed to 1 and 10μM dexamethasone alone versus 28.6±2.8% and 25 ±1.9% for those supplemented with 2μM resveratrol (Fig 5A) respectively. Also, the expression level of the p53 and p21 genes was measured by qPCR 24 hours after dexamethasone removal. Consistent with our earlier experiments, p53 and p21 were upregulated in dexamethasone-treated cells at both concentrations. The level of p53 and p21 message was significantly reduced in tenocytes co-treated with 2μM resveratrol compared to the dexamethasone-treated groups (p<0.05, p<0.01 Fig 5B).

**Fig 6:**
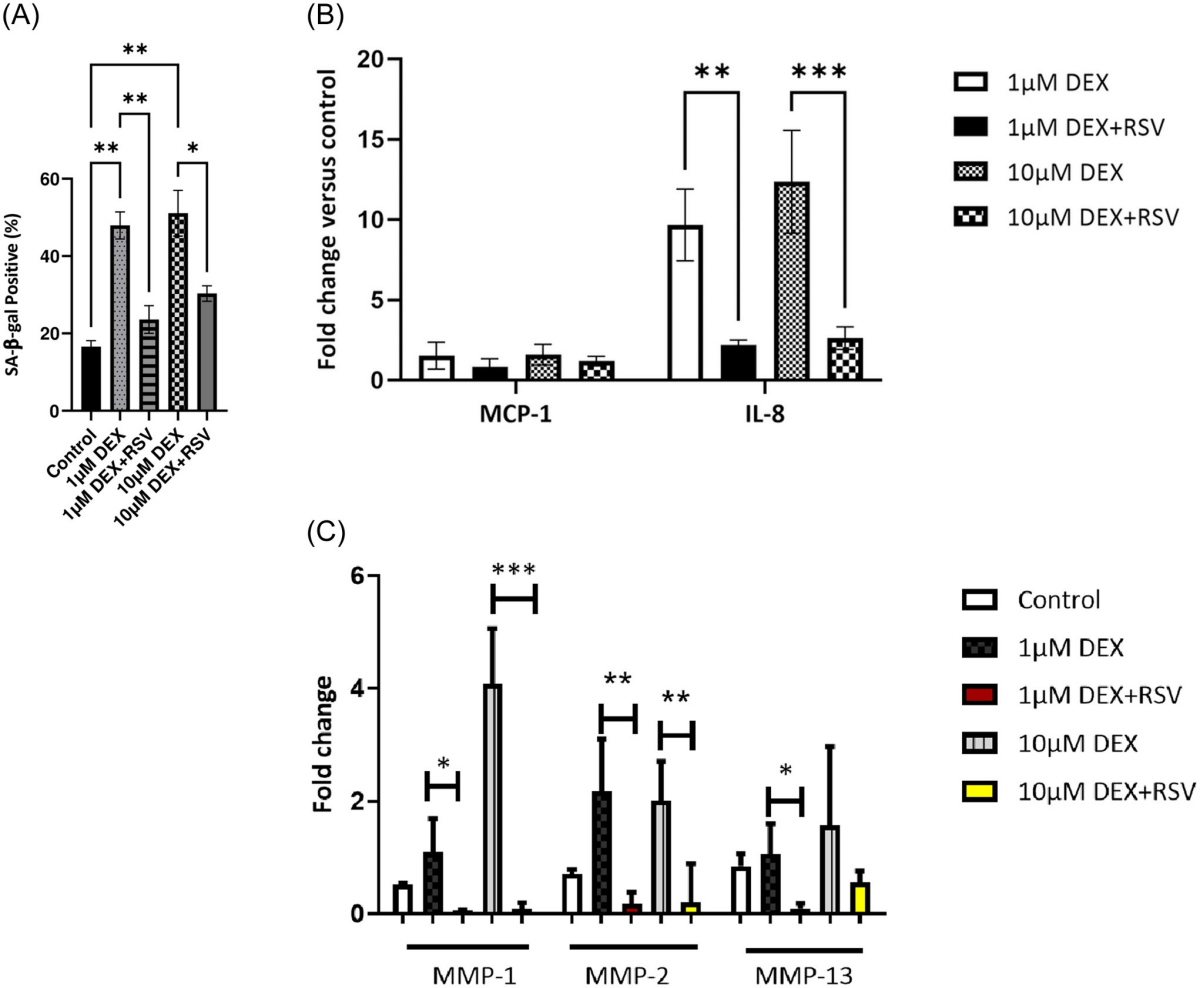
**A.** Quantification of SA-β-gal staining following a 48-hour exposure to 1 and 10μM dexamethasone and 2μM resveratrol (DEX+RSV). Cells were assessed for the percentage of SA-β-gal positive staining seven days post-treatment. SA-β-gal staining was performed in duplicate and at least 100 cells were inspected to determine the percentage of SA-β-gal positive cells. Statistical analysis performed using One-way ANOVA (*p<0.05,**p< 0.01). **B.** Resveratrol inhibited SASP development in dexamethasone-treated cells. The levels of IL-8 and MCP-1 were measured by cytokine array 5 days after treatment with 1 and 10μM dexamethasone and 2μM resveratrol (DEX+RSV). The values are shown as the mean ± SD. **p<0.01,***p < 0.001. **C.** The effect of resveratrol on MMPs gene expression in dexamethasone and resveratrol-treated (DEX+RSV) TDCs. The gene expression levels of MMP-1, MMP-2, and MMP-13 were measured by qPCR five days after the removal of treatment. Data were normalized to GAPDH expression and presented as fold change over the control level. Statistical analysis performed using One-way ANOVA (*p<0.05, **p< 0.01, ***p<0.001).

The activity of cellular senescence biomarker SA-β-Gal at pH 6.0 was measured 7 days after treatment with 1 and 10μM dexamethasone and 2μM resveratrol. As shown in Fig 6A, the percentage of SA-β-Gal positive cells increased significantly in cultures treated with 1 and 10μM dexamethasone-treated cells compared to non-treated cells (p< 0.01). However, in cultures treated with 2μM resveratrol alongside 1 and 10μM dexamethasone, there was a notable reduction in the percentage of β-gal positive cells (p < 0.01, p < 0.05 respectively), indicating a protective effect against the induction of senescence by dexamethasone.

Induction of senescence by dexamethasone resulted in SASP development in equine tenocytes which comprised an upregulation of MCP-1 and IL-8 at the protein level after 5 days. Therefore, to determine if resveratrol treatment supressed the SASP the protein levels of MCP-1 and IL-8 were measured in dexamethasone and resveratrol-treated tenocytes. As shown in Fig 6B, the secretion of IL-8 was significantly reduced when cells were co-treated with resveratrol and dexamethasone at both concentrations (p<0.01 and p<0.001, Fig 6B).

As in previous experiments, levels of MMP-1, MMP-2, and MMP-13 increased in cultures treated with dexamethasone. However, the levels of MMP-1 and MMP-2 reduced significantly in cultures treated with both 1 and 10μM dexamethasone, as well as 2μM resveratrol (Fig 6C; p < 0.05, p < 0.01, p < 0.001). Furthermore, MMP-13 gene expression decreased significantly in cells treated with 1μM dexamethasone and resveratrol compared to 1μM dexamethasone treated cultures (p < 0.05). These data suggesting a potential suppressive effect of resveratrol on MMPs expression.

### SIRT1 dependency/independency

**Fig 7:**
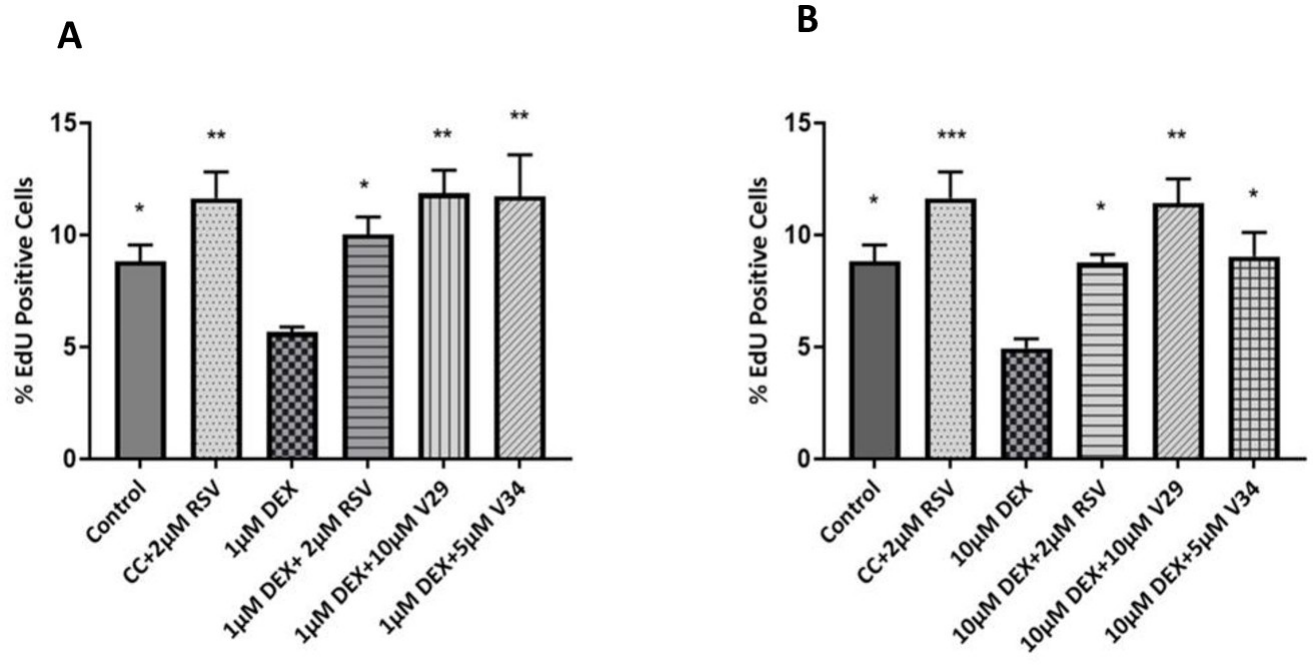
EdU labelling for proliferating cells. Statistically significant differences between 1 (A) or 10μM dexamethasone (DEX) (B) and Resveratrol (RSV)/ V34 and V29+ DEX are indicated by * p<0.05 ** p<0.01.

**Fig 8:**
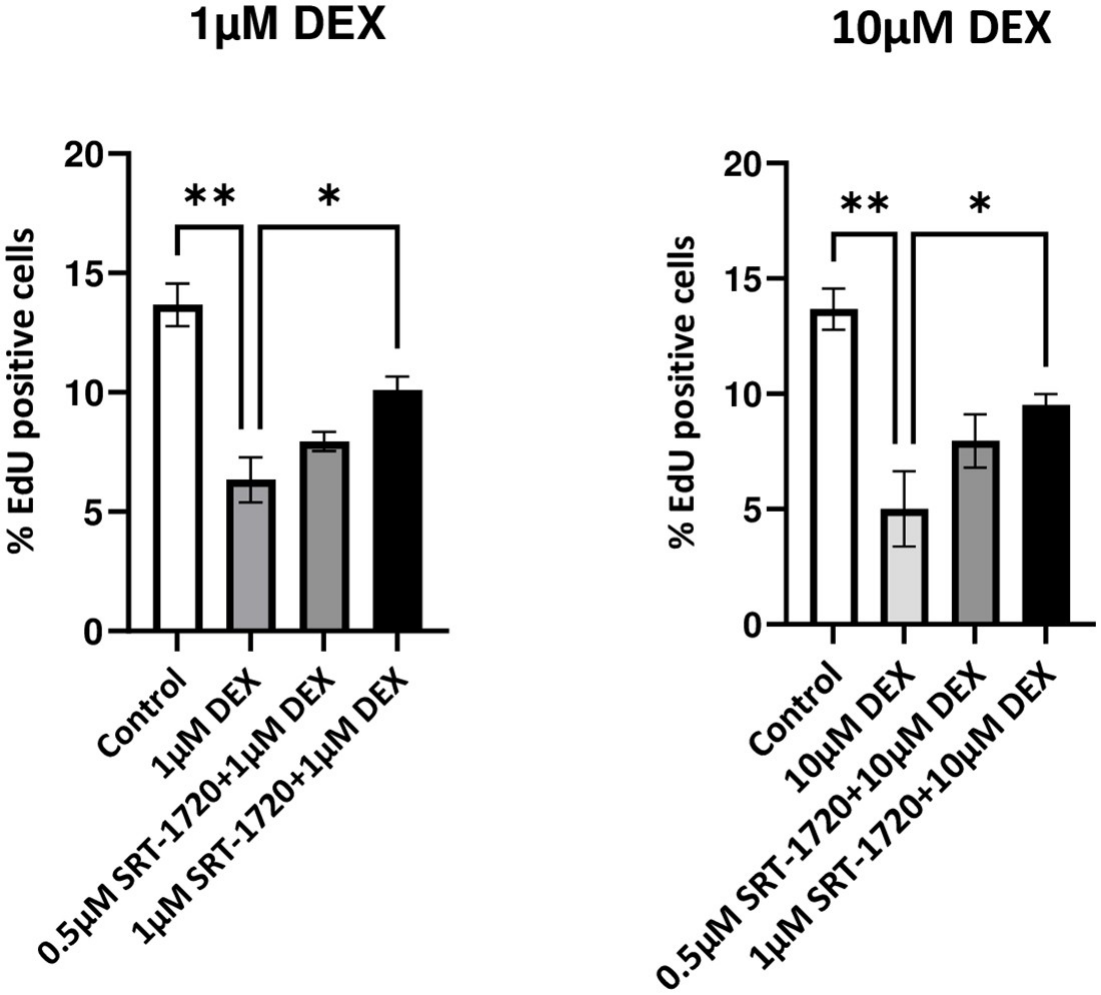
The effect of different concentrations of SRT-1720 on TDCs proliferation against dexamethasone treatment. Data are presented as the Mean ± SD of EDU positive cells observed between 1 and 10μM dexamethasone (DEX) and 0.5 and 1μM SRT-1720 (SIRT). Statistical analysis was performed using One-Way ANOVA with Bonferroni’s Multiple Comparison Test (*p<0.05, **p< 0.01) (n = 3).

To understand the potential mechanism of action by which protection from senescence induced by dexamethasone occurs, tenocytes were co-treated with resveratrol and structural series of novel Resveralogues including 5μM V34 and 10μM V29 plus 1 and 10μM dexamethasone for 48 hours. These differ both in their endogenous antioxidant capacities and ability to activate SIRT1, a canonical target of resveratrol. As shown in Fig 7A and 7B all the resveralogues were able to protect the cells from SIS. Although protection was lower when cells were co-treated with 10μM dexamethasone plus V34 this was still highly significant (p<0.05. Fig 7B). Treatment with the potent SIRT1 activator SRT-1720 also significantly protected equine tenocytes from SIS but to a somewhat lower degree than any of the resveralogues (Fig 8).

## Discussion and conclusion

It has been shown that human tenocytes will enter a senescent state within 48 to 72 hours of treatment with micromolar concentrations of dexamethasone *in vitro* and *in vivo* and that senescence is associated with significant shifts in phenotype including the upregulation of the p53/p21 pathway. We have demonstrated, for the first time that exposure to corticosteroids also triggers SIS in equine tenocytes. Equine SIS is associated with upregulation of the classical cyclin dependent kinase inhibitors associated with entry into the senescent state as well as a shift to a catabolic phenotype marked by upregulation of selected pro-inflammatory cytokines and matrix degrading enzymes.

The pathological implications of the accumulation of such cells within a tendon whether through natural cell turnover or as a side effect of steroid treatment are obvious and troubling. Previous studies in equine tendons have suggested that senescent tenocytes potentially increase the susceptibility of a tendon to degenerative tendinopathy as a result of increases in MMP expression. Decreased collagen production and altered MMP expression (particularly an upregulation of MMP13 levels) are observed in senescent tenocytes in horses which match age-related changes described in cyclically loaded tendons in *vitro* [13]. Thus, since senescent cells are causal agents of “sterile inflammation” their accumulation by any route will drive tendon degeneration and impair tendon healing.

Fortunately, as in humans, we have shown that SIS can be prevented using resveratrol at doses in the low micromolar range. Although resveratrol has poor bioavailability these concentrations are clinically achievable either by dietary means or through injection [14] opening up the possibility that a combination of corticosteroid and resveratrol treatment would allow the suppression of inflammation within a tendon without SIS and improve healing.

For it to become a useful enhancement of existing therapies it is important to understand the mechanism by which resveratrol prevents SIS. The time taken for corticosteroids to induce a senescent state in tenocytes is relatively short, which is not, at first sight, easy to reconcile with models of senescence induction based on damage to telomeres and accelerated end-replication loss (which would require passage through S phase and cell division). An important and informative earlier study [4] showed that over expression of SIRT1, the canonical target of resveratrol, is also protective against SIS. This implied that the mechanism by which resveratrol protects against SIS requires SIRT1 activation. To test this, we exposed equine tenocytes to dexamethasone and employed the SIRT1 activator SRT-1720 as an alternative protective compound. SRT-1720 is a specific and far more potent activator of SIRT1 than resveratrol (EC_1.5_ = 0.16 μM compared to 46.2 μM for resveratrol [15]) but it did not protect tenocytes from SIS as effectively as resveratrol. In contrast, several of our novel resveralogues were as protective against an identical challenge with dexamethasone as resveratrol. This included V29, a resveratrol analogue with no capacity to activate SIRT1. These observations are inconsistent with a simple hypothesis in which protection from SIS is purely SIRT1-dependent and suggests that, as with rescue from telomere-dependent senescence in human cells, a combination of SIRT1 dependent and independent processes contribute to the protective effects of resveralogues in equine tenocytes.

Our use of a compound series also allows us a supplementary insight into the mechanisms of SIS [4, 16] considered the, not unreasonable, possibility that simple chemical scavenging of reactive oxygen species (ROS) by resveratrol might be an important protective mechanism (as evidenced by their demonstration of protection against SIS using vitamin C). However, the endogenous radical scavenging capacities of the resveralogues we have used in this study vary significantly across the structural series (some lack the phenolic functionality essential to radical scavenging [8]) yet all of them protect equally against SIS. This implies that ROS generation is a relatively minor aspect of the senescence induction process in this system. With this is mind, and until the mechanisms of SIS are better understood, caution is probably warranted in any use of vitamin C as an adjunct to corticosteroid treatment since the molecule can act as a potent pro-oxidant under certain conditions [17].

Thus, understanding the mechanism by which SIS occurs and by which these various compounds protect is an important next step in identifying or developing molecules which can activate both SIRT1 dependent and independent pathways and which have better bioavailability than resveratrol. This work has not only demonstrated further the adverse effects of corticosteroids on tenocytes, but also that resveralogues may offer the prospect of mitigating some of these adverse effects and also provide a means to inhibit age- and exercise- related senescence, thereby helping reduce the incidence of tendinopathy and enhancing healing.

## Supporting information

S1 Fig Effect of V29 at different concentrations on TDCs proliferation. Dose-Dependent Response of V29 on tenocytes proliferation was assessed. TDCs were treated with different concentrations of V29 (2, 5,10 and 15μM) for 24 hours, followed by EdU labeling to measure the total cycling fraction of cells under different dosages.
(TIF)

S2 Fig Effect of SIRT-1720 at different concentrations on TDCs proliferation. Tenocytes were treated with 0.2, 0.5, 1 and 2μM concentration of SIRT-1720 for 24h, followed by EdU labelling to measure the total cycling fraction of cells.
(TIF)

S3 Fig Results obtained with RayBio Equine (horse) cytokine array 1. These membranes were probed with media from 1 and 10μM dexamethasone treated (DEX) and control cells.
(TIF)

S4 Fig Effect of V34 at different concentrations on TDCs proliferation. Dose-Dependent Response of V34 on TDCs Proliferation. TDCs were treated with different concentrations of V34 for 24 hours, followed by EdU labeling to assess the total cycling fraction of cells under different dosages. The data reveal a dose-dependent relationship between V34 concentration and the proliferative response of tenocytes.
(TIF)

S5 Fig Resveratrol dose response. To investigate the effect of resveratrol on tenocytes proliferation, an EdU assay was performed. Cells were treated with different concentrations of resveratrol (2, 5, 10, 15,20 and 30 μM) for a 24-hour incubation period.
(TIF)

S6 Fig SIRT1 gene expression. SIRT-1 gene expression analysis post-treatment with 1μM SIRT-1720, 2μM resveratrol (RSV), and 10μM V29, with or without 1 and 10μM dexamethasone (DEX). Control cultures were maintained in medium only. RNA extraction was conducted immediately post-treatment removal. SIRT-1 gene expression is significantly upregulated in cultures treated with SIRT-1720 and resveratrol, while it remained unexpressed following V29 treatment. Conversely, SIRT-1 expression was downregulated following 1 and 10μM dexamethasone treatment. Data normalization was based on GAPDH expression and represented as fold change relative to the control level. Statistical significance was determined using One-way ANOVA (****p < 0.0001).
(TIF)

S7 Fig EdU and Ki67 staining. Proliferating cells were labelled with EdU (red) and ki67 (green). Cell nuclei were stained with DAPI (blue). Images were taken with a fluorescent microscope at 20X magnification. Scale bar 400μm.
(TIF)

S8 Fig SA-β-Gal staining. SA-β-Gal staining following 7 days after a 48-hour exposure to l and 10 μM dexamethasone and resveratrol. (A: non-treated cells, B: 1 μM dexamethasone-treated cells, C: 10 μM dexamethasone-treated cells, D: Resveratrol+1 μM dexamethasone-treated cells, E: Resveratrol+10 μM dexamethasone-treated cells).
(TIF)

S9 Fig Effect of DMSO on the proliferation of control cells compared with etoposide treatment. Cells were treated with either DMSO (<1%) or 10 μM etoposide for a duration of 48 hours. Following the treatment period, EdU labeling was performed to measure the cell proliferation. Statistical significance was determined using a One-way ANOVA (***p < 0.001).
(TIF)

S10 Fig Percentage of cycling cells relative to control. Low dose SRT-1720 (0.5μM) increased the proliferating fraction of 1 and 10μM DEX treated cells from 46 and 36% to about 58 percent respectively. 1μM SRT-1720 treatment increased the proliferation fraction of 1 and 10μDEX treated cells to about 74 and 69.5% respectively. However, resveralogoues compound specifically V29 increased the proliferation fraction from 64 and 56% in 1 and 10μM DEX treated cells to about 134 and 129.5% respectively. Resveralogues show a significantly greater protective effect against senescence induced by DEX treatment compared to SRT-1720. Specifically, V29 increases the proliferating fraction of DEX-treated cells to a much higher extent, suggesting a better capacity to mitigate the senescence-inducing effects of DEX.
(TIF)

## References

[1] Dakin S.G., Dudhia J., and Smith R.K., Resolving an inflammatory concept: the importance of inflammation and resolution in tendinopathy. Vet Immunol Immunopathol, 2014. 158(3–4): p. 121–7. doi: 10.1016/j.vetimm.2014.01.007 24556326 PMC3991845

[2] Dakin S.G., et al., Proresolving Mediators LXB4 and RvE1 Regulate Inflammation in Stromal Cells from Patients with Shoulder Tendon Tears. Am J Pathol, 2019. 189(11): p. 2258–2268. doi: 10.1016/j.ajpath.2019.07.011 31437425 PMC6876268

[3] Dakin S.G., et al., Inflammation activation and resolution in human tendon disease. Sci Transl Med, 2015. 7(311): p. 311ra173. doi: 10.1126/scitranslmed.aac4269 26511510 PMC4883654

[4] Poulsen R.C., Glucocorticoids induce senescence in primary human tenocytes by inhibition of sirtuin 1 and activation of the p53/p21 pathway: in vivo and in vitro evidence. 2014. 10.1136/annrheumdis-2012-203146 PMC4078757 23727633

[5] Faragher R.G., et al., Senescence in the aging process. F1000Res, 2017. 6: p. 1219. doi: 10.12688/f1000research.10903.1 28781767 PMC5531163

[6] Faragher R.G.A., Heidari N., and Ostler E.L., Therapeutic Opportunities Presented by Modulation of Cellular Senescence. Subcell Biochem, 2023. 102: p. 175–193. doi: 10.1007/978-3-031-21410-3_8 36600134

[7] Dudhia J., et al., Aging enhances a mechanically-induced reduction in tendon strength by an active process involving matrix metalloproteinase activity. Aging Cell, 2007. 6(4): p. 547–56. doi: 10.1111/j.1474-9726.2007.00307.x 17578513

[8] Birar V.C., et al., A facile, stereoselective, one-pot synthesis of resveratrol derivatives. Chemistry Central journal, 2015. 9(1): p. 26–26. doi: 10.1186/s13065-015-0102-7 26023318 PMC4446909

[9] Latorre E., et al., Small molecule modulation of splicing factor expression is associated with rescue from cellular senescence. BMC cell biology, 2017. 18(1): p. 31–15. doi: 10.1186/s12860-017-0147-7 29041897 PMC5645932

[10] Kelly E., et al., Science-in-brief: The importance of senescence in tendinopathy: New opportunities. Equine Vet J, 2020. 52(3): p. 349–351. doi: 10.1111/evj.13228 32259376

[11] Barsby T. and Guest D., Transforming growth factor beta3 promotes tendon differentiation of equine embryo-derived stem cells. Tissue Eng Part A, 2013. 19(19–20): p. 2156–65. doi: 10.1089/ten.TEA.2012.0372 23611525

[12] Birar V.C., et al., Novel resveratrol derivatives have diverse effects on the survival, proliferation and senescence of primary human fibroblasts. Biogerontology, 2020. 21(6): p. 817–826. doi: 10.1007/s10522-020-09896-6 32793997

[13] Smith R.K., et al., The influence of ageing and exercise on tendon growth and degeneration—hypotheses for the initiation and prevention of strain-induced tendinopathies. Comp Biochem Physiol A Mol Integr Physiol, 2002. 133(4): p. 1039–50. doi: 10.1016/s1095-6433(02)00148-4 12485691

[14] Kim D.H., et al., Change in plasma sirtuin 1 level by injection into uterus of resveratrol in Korean cattle. Anim Reprod, 2020. 17(1): p. e20190090. doi: 10.21451/1984-3143-AR2019-0090 32399068 PMC7212746

[15] Milne J.C., et al., Small molecule activators of SIRT1 as therapeutics for the treatment of type 2 diabetes. Nature, 2007. 450(7170): p. 712–6. doi: 10.1038/nature06261 18046409 PMC2753457

[16] Poulsen R.C., Carr A.J., and Hulley P.A., Protection against glucocorticoid-induced damage in human tenocytes by modulation of ERK, Akt, and forkhead signaling. Endocrinology, 2011. 152(2): p. 503–14. doi: 10.1210/en.2010-1087 21209015

[17] Pavey K.D., et al., Vitamin C induced decomposition of lipid hydroperoxides: direct evidence of genotoxin-DNA binding detected by QCRS. Chem Commun (Camb), 2001(18): p. 1886–7. doi: 10.1039/b105766a 12240363

