# Supplementary figures and images for: A SIRT1-independent mechanism mediates protection against steroid-induced senescence by resveralogues in equine tenocytes
Source: PLoS One. 2024 Aug 22;19(8):e0309301. doi: 10.1371/journal.pone.0309301 (PMC11340939; doi:10.1371/journal.pone.0309301)

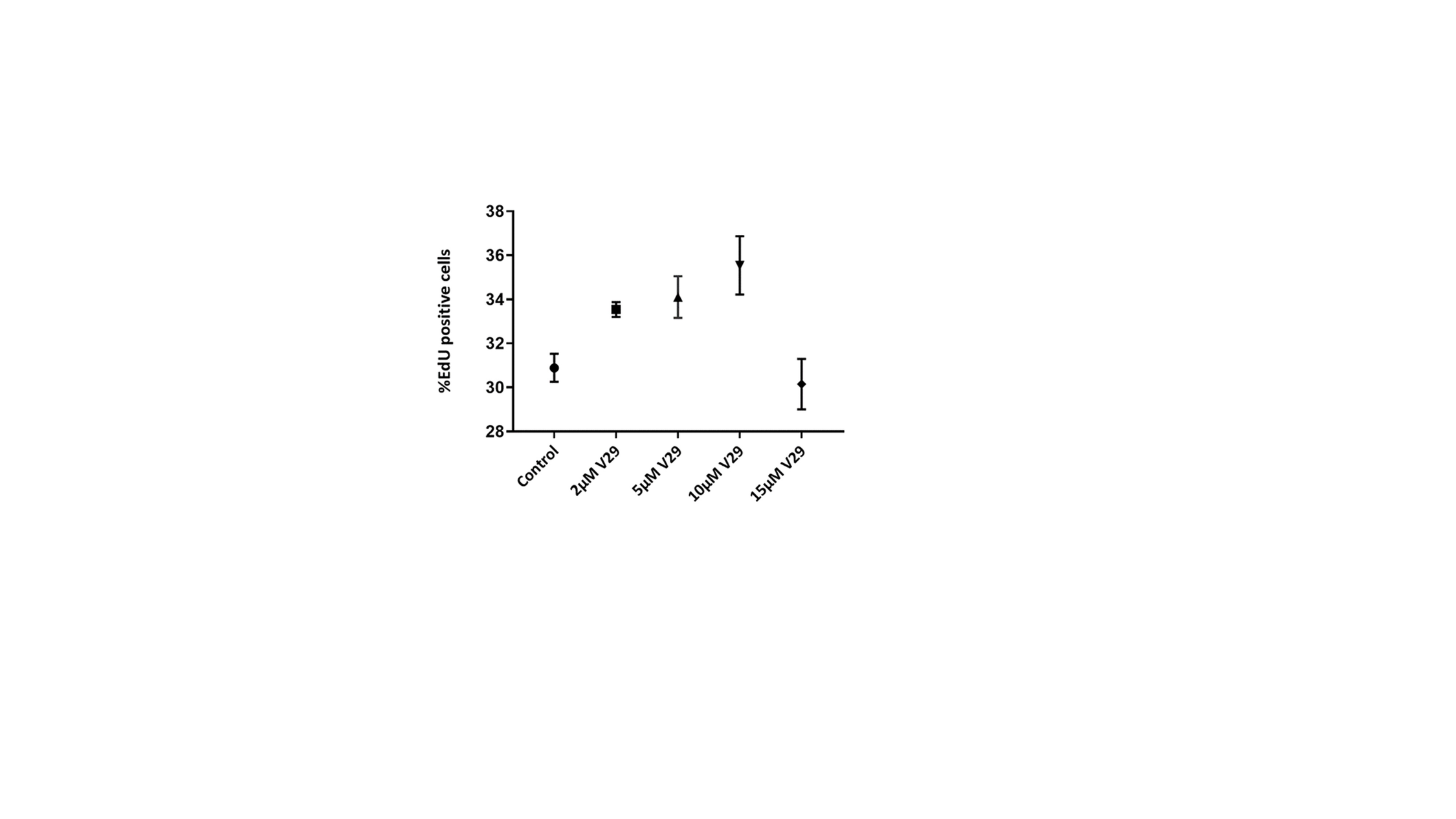

Supplement: S1 Fig — Dose-Dependent Response of V29 on tenocytes proliferation was assessed. TDCs were treated with different concentrations of V29 (2, 5,10 and 15μM) for 24 hours, followed by EdU labeling to measure the total cycling fraction of cells under different dosages. (TIF) [file pone.0309301.s001.tif]

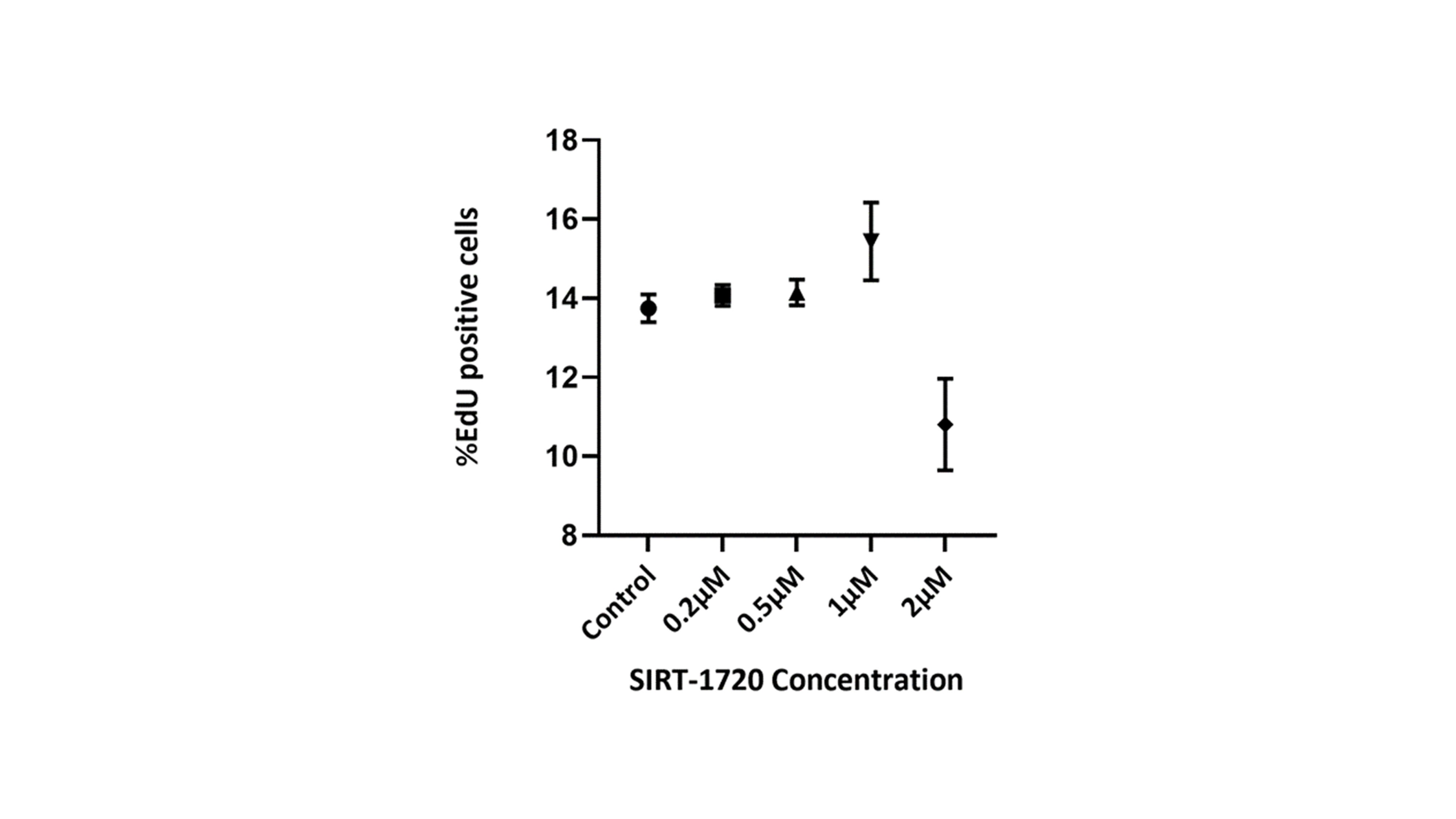

Supplement: S2 Fig — Tenocytes were treated with 0.2, 0.5, 1 and 2μM concentration of SIRT-1720 for 24h, followed by EdU labelling to measure the total cycling fraction of cells. (TIF) [file pone.0309301.s002.tif]

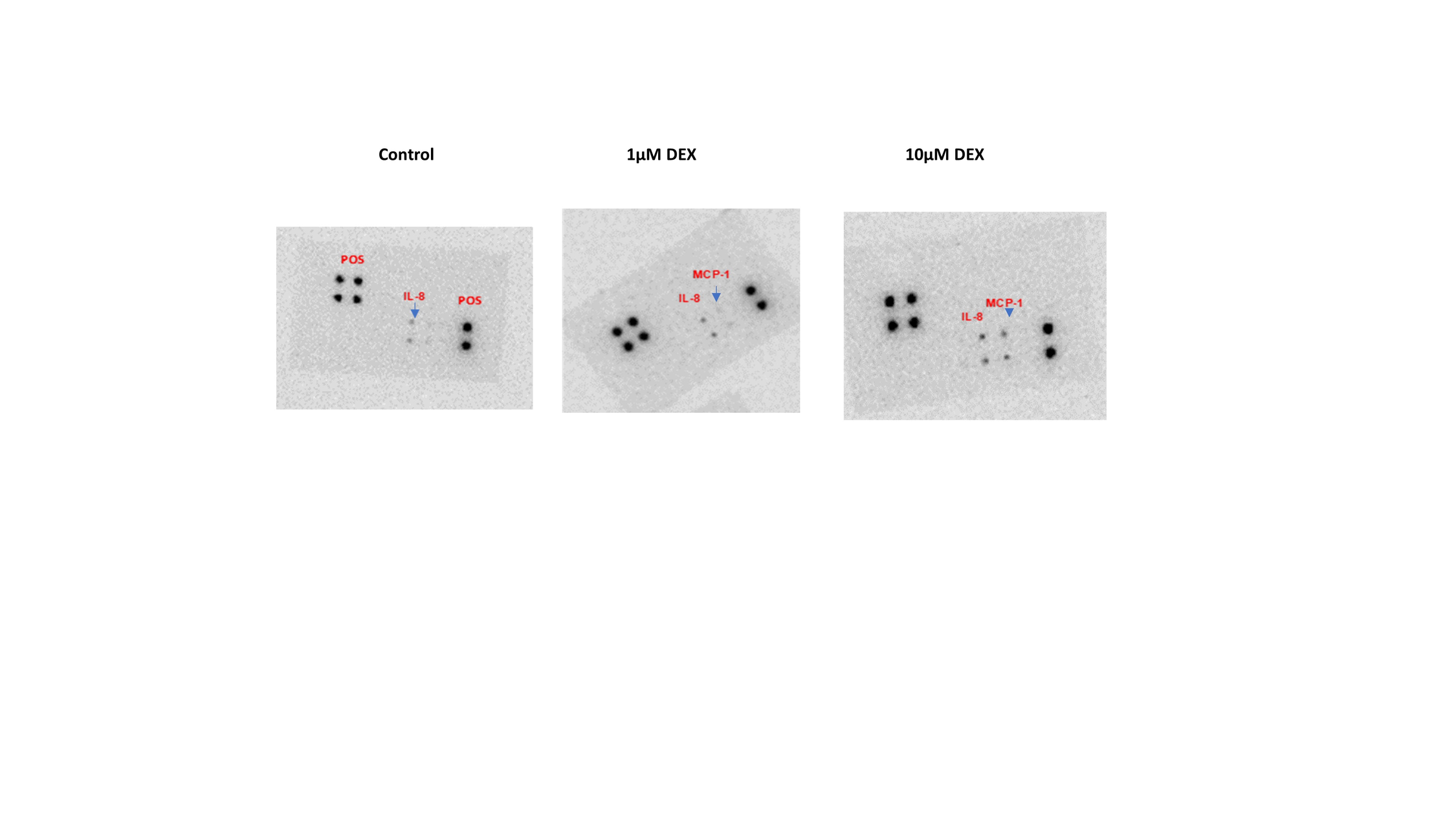

Supplement: S3 Fig — These membranes were probed with media from 1 and 10μM dexamethasone treated (DEX) and control cells. (TIF) [file pone.0309301.s003.tif]

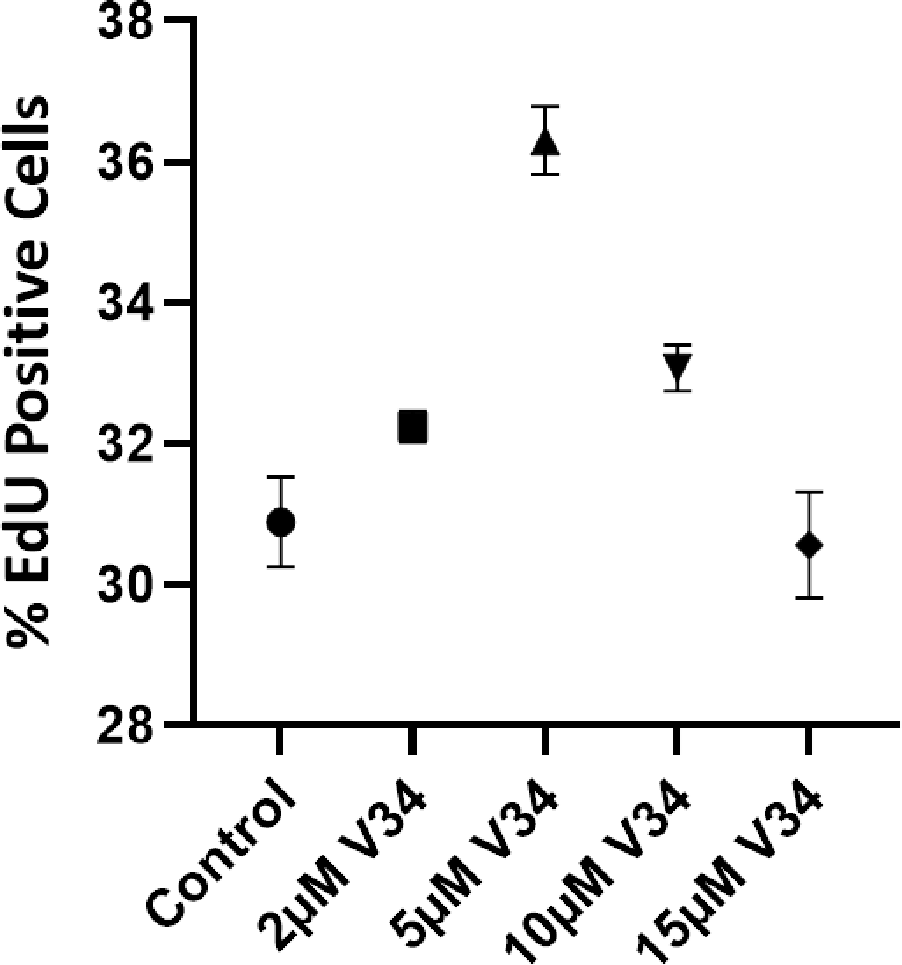

Supplement: S4 Fig — Dose-Dependent Response of V34 on TDCs Proliferation. TDCs were treated with different concentrations of V34 for 24 hours, followed by EdU labeling to assess the total cycling fraction of cells under different dosages. The data reveal a dose-dependent relationship between V34 concentration and the proliferative response of tenocytes. (TIF) [file pone.0309301.s004.tif]

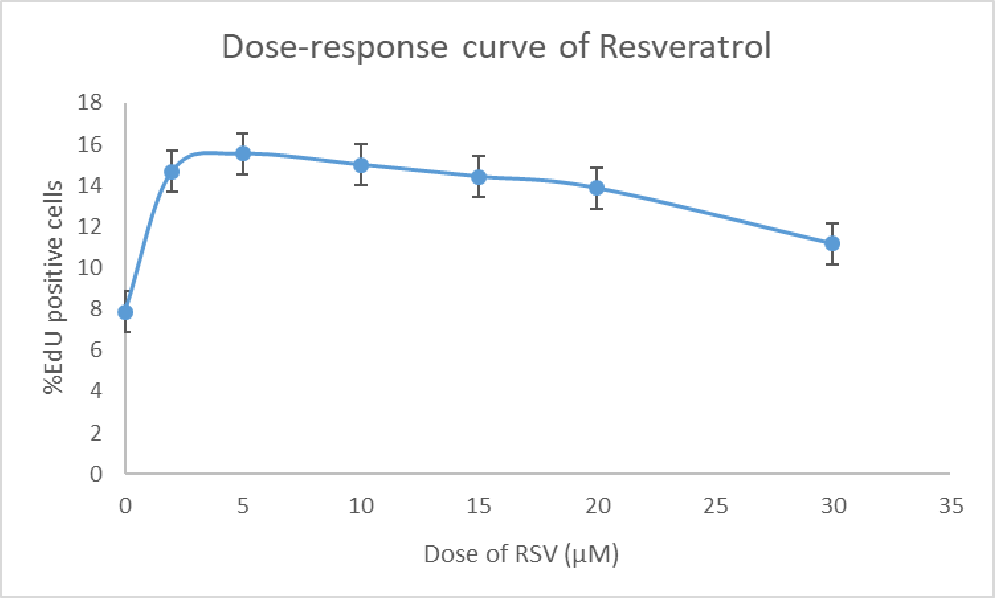

Supplement: S5 Fig — To investigate the effect of resveratrol on tenocytes proliferation, an EdU assay was performed. Cells were treated with different concentrations of resveratrol (2, 5, 10, 15,20 and 30 μM) for a 24-hour incubation period. (TIF) [file pone.0309301.s005.tif]

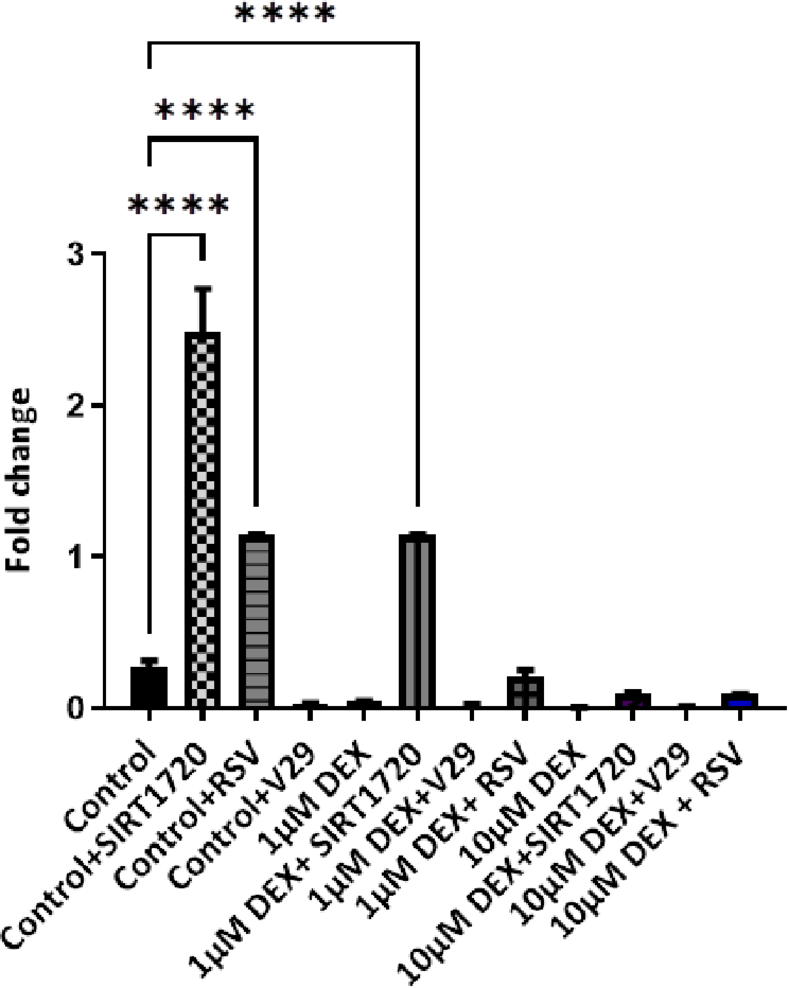

Supplement: S6 Fig — SIRT-1 gene expression analysis post-treatment with 1μM SIRT-1720, 2μM resveratrol (RSV), and 10μM V29, with or without 1 and 10μM dexamethasone (DEX). Control cultures were maintained in medium only. RNA extraction was conducted immediately post-treatment removal. SIRT-1 gene expression is significantly upregulated in cultures treated with SIRT-1720 and resveratrol, while it remained unexpressed following V29 treatment. Conversely, SIRT-1 expression was downregulated following 1 and 10μM dexamethasone treatment. Data normalization was based on GAPDH expression and represented as fold change relative to the control level. Statistical significance was determined using One-way ANOVA (****p < 0.0001). (TIF) [file pone.0309301.s006.tif]

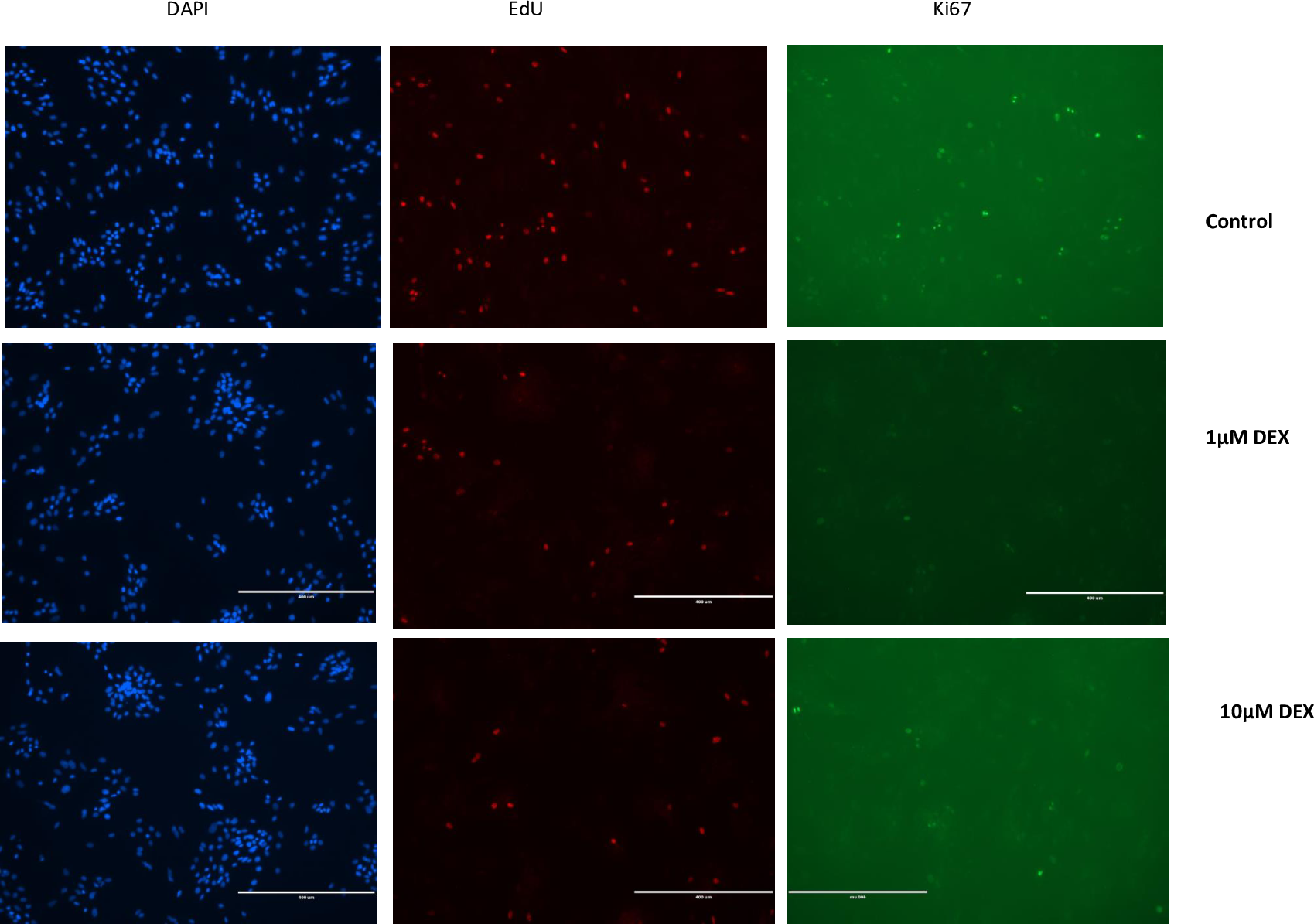

Supplement: S7 Fig — Proliferating cells were labelled with EdU (red) and ki67 (green). Cell nuclei were stained with DAPI (blue). Images were taken with a fluorescent microscope at 20X magnification. Scale bar 400μm. (TIF) [file pone.0309301.s007.tif]

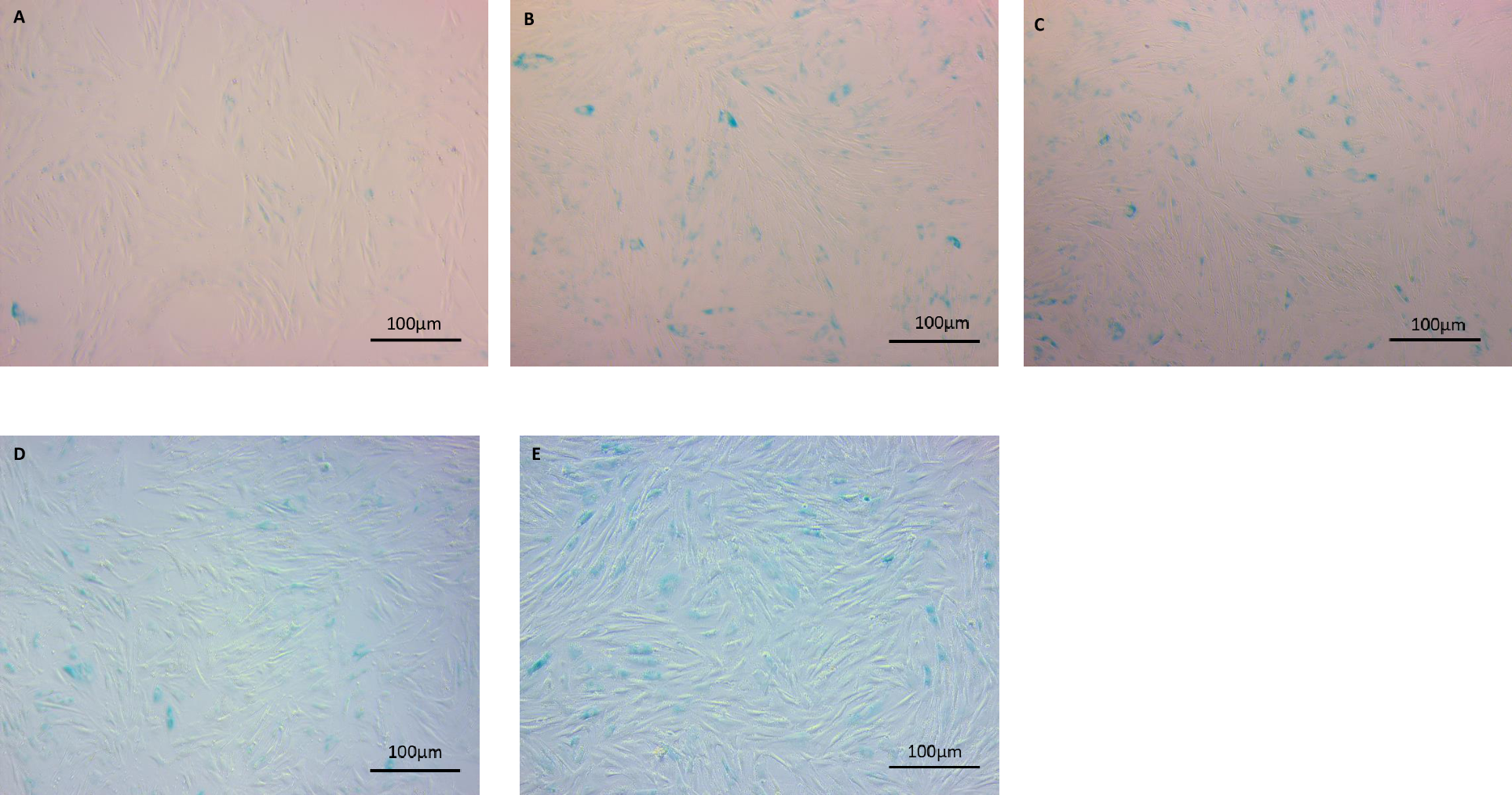

Supplement: S8 Fig — SA-β-Gal staining following 7 days after a 48-hour exposure to l and 10 μM dexamethasone and resveratrol. (A: non-treated cells, B: 1 μM dexamethasone-treated cells, C: 10 μM dexamethasone-treated cells, D: Resveratrol+1 μM dexamethasone-treated cells, E: Resveratrol+10 μM dexamethasone-treated cells). (TIF) [file pone.0309301.s008.tif]

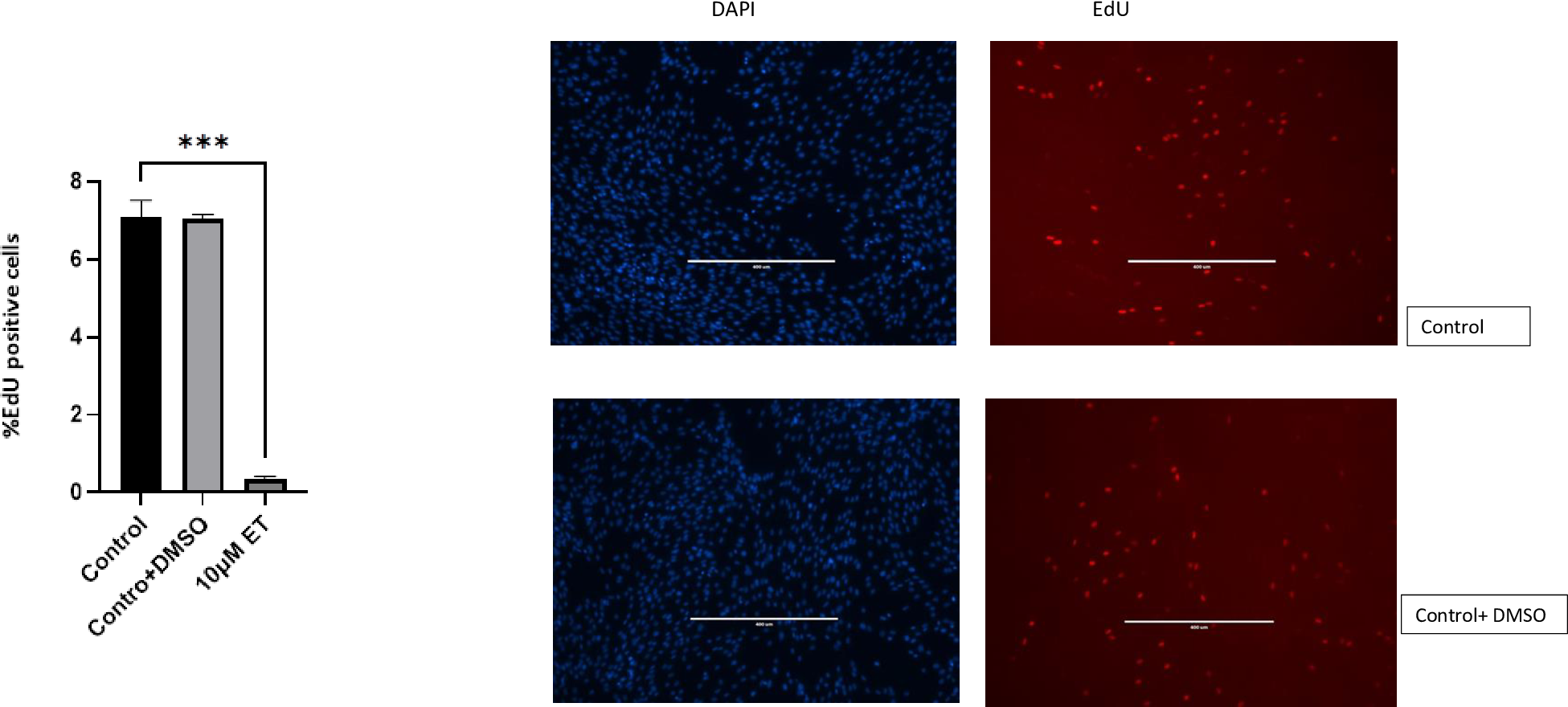

Supplement: S9 Fig — Cells were treated with either DMSO (<1%) or 10 μM etoposide for a duration of 48 hours. Following the treatment period, EdU labeling was performed to measure the cell proliferation. Statistical significance was determined using a One-way ANOVA (***p < 0.001). (TIF) [file pone.0309301.s009.tif]

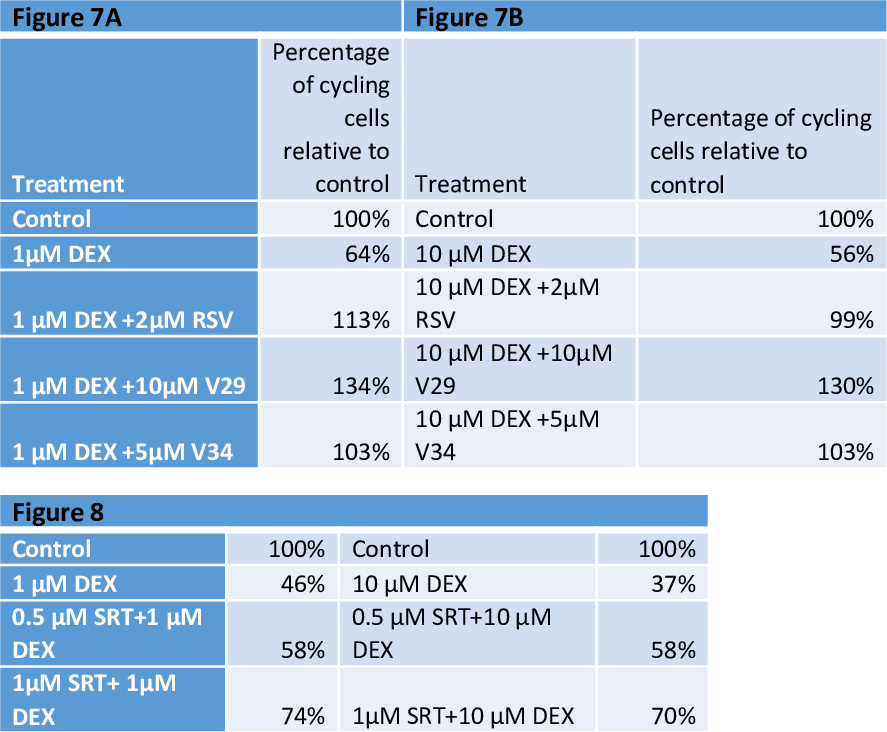

Supplement: S10 Fig — Low dose SRT-1720 (0.5μM) increased the proliferating fraction of 1 and 10μM DEX treated cells from 46 and 36% to about 58 percent respectively. 1μM SRT-1720 treatment increased the proliferation fraction of 1 and 10μDEX treated cells to about 74 and 69.5% respectively. However, resveralogoues compound specifically V29 increased the proliferation fraction from 64 and 56% in 1 and 10μM DEX treated cells to about 134 and 129.5% respectively. Resveralogues show a significantly greater protective effect against senescence induced by DEX treatment compared to SRT-1720. Specifically, V29 increases the proliferating fraction of DEX-treated cells to a much higher extent, suggesting a better capacity to mitigate the senescence-inducing effects of DEX. (TIF) [file pone.0309301.s010.tif]
